# Supplementary material for: Coherent multidimensional spectroscopy of dilute gas-phase nanosystems
Source: Nat Commun. 2018 Nov 16;9:4823. doi: 10.1038/s41467-018-07292-w (PMC6240067; doi:10.1038/s41467-018-07292-w)
Supplement: Supplementary file 1 — Supplementary Information [file 41467_2018_7292_MOESM1_ESM.pdf]

Supplementary Information:

Coherent multidimensional spectroscopy of dilute gas-phase nanosystems

Lukas Bruder[1]*, Ulrich Bangert[1], Marcel Binz[1], Daniel Uhl[1], Romain Vexiau[2], Nadia Bouloufa-Maafa[2], Olivier Dulieu[2] and Frank Stienkemeier[1]

[1]Institute of Physics, University of Freiburg, Hermann-Herder-Str. 3, 79104 Freiburg, Germany
[2]Laboratoire Aimé Cotton, CNRS, Université Paris-Sud, ENS Cachan, Université Paris-Saclay, 91405 Orsay Cedex, France
*Correspondence to: lukas.bruder@physik.uni-freiburg.de

Supplementary Note 1: Optical setup and data acquisition:

The 2DES setup is shown in Supplementary Fig. 2. In a threefold nested optical interferometer, a collinear four-pulse train is generated, with pulse delays, denoted τ, t (coherence times) and T (evolution time), each controlled with a motorized translation stage (Supplementary Fig. 2 and Fig 1b). An acousto-optical modulator (AOM), installed in each interferometer arm, imprints a quasi-continuous phase modulation (modulation frequency Ω_i) onto the transmitted laser pulses. The pulse train is focused (f=300 mm) into the vacuum apparatus where it intersects with the helium droplet beam. Created photoelectrons/-ions are deflected by two electrodes and detected with a channeltron detector. The signal is amplified and fed into a lock-in amplifier (LIA) (MFLI, Zürich Instruments).

The four phase-modulated laser pulses induce a third-order nonlinear polarization into the sample (pulse 1-3), which is mapped onto a population state (pulse 4) and detected in the electron/ion yield upon photoionization. Double-sided Feynman diagrams schematically show this process (Supplementary Fig. 3). Phase-locked driving of the AOMs at relative frequencies $\Omega_2 - \Omega_1 = \Omega_{21} = 5$ kHz and Ω_{43}= 8 kHz leads to characteristic well-defined modulations of the nonlinear signal contributions. This allows for efficient extraction of the rephasing (RP, photon echo) and non-rephasing (NRP) 2D signals at the difference- ($\Omega_{\mathrm{diff}} = \Omega_{43} -$

$\Omega_{21} = 3$ kHz, RP pathways) and sum-frequency sidebands ($\Omega_{\mathrm{sum}} = \Omega_{43} + \Omega_{21} = 13$ kHz, NRP pathways), respectively using lock-in amplification.

At beam splitters 4 and 5, a copy of the phase-modulated pulses is branched off and fed into two monochromators (IHR550, Horiba Scientific) to yield a 3 and 13 kHz-reference signals for phase-synchronous lock-in detection upon which real and imaginary parts of the RP and NRP signals are simultaneously detected. Amplitude and phase information are hence retrieved through heterodyned lock-in detection. In addition, phase noise, picked up in the optical interferometers, appears correlated in the signal and reference and therefore efficiently cancels in the demodulation process, leading to a passive phase stabilization of the interferometric measurements.

Eventually, the sum of RP and NRP signals yields the complex-valued 2D response function $S(\tau,T,t)$. A 2D Fourier transform with respect to τ and t returns the 2D frequency-correlation maps $\tilde{S}(\omega_\tau, T, \omega_t)$ of which the real part (absorption spectrum) is shown in the main text. The retrieved spectra directly correlate the frequency-resolved pump (ω_τ-axis) and probe response of the system (ω_t-axis) as parametric function of the evolution time T.

Supplementary Fig. 3 shows exemplary the RP pathways as induced in the experiment and extracted by the phase modulation. GSB pathways propagate on the electronic ground state while SE and ESA pathways propagate on the excited electronic state manifold. The absolute magnitude of these signals reflects the detected photoelectron/-ion count rates contributing to the individual pathways. However, the phase of each feature arises from heterodyned detection of the nonlinear signals with a reference waveform, which is done in the lock-in amplifier. This leads to a positive amplitude of SE/GSB pathways and a negative amplitude of ESA pathways as shown in Supplementary Fig. 3. Note, that in collinear action-signal based 2DES always two destructively interfering ESA pathways exist, which is in contrast to non-collinear phase-matching schemes[1]. Since in our experiments both ESA pathways scale differently with laser intensity (Supplementary Fig. 3), we obtain consistently a negative

net contribution, which may not be the case in fluorescence-detected 2DES[1] and is an advantage of our scheme.

Correct phasing of the 2D spectra is crucial and often a technical issue in 2DES[2]. As an advantage of the phase modulation approach, phasing is readily done by adjusting a reference phase in the lock-in amplifier as described in Ref. 3. In our photoionization experiments we cross-checked the correct phasing with 2DES measurements of atomic Rb in an effusive atomic beam which provides us simplified, particularly sharp 2D spectra of which the phase behavior is well-known.

Measurements are done with a four- or five-pulse scheme. For the latter, a fifth pulse (not phase-modulated) is collinearly superimposed with a delay of $\Delta \approx 2$ ns using a polarizing beam splitter to minimize losses. Independent wavelength-tuning of this pulse allows selection of certain ionization channels to enhance individual signal components. In case of photoion detection, mass resolution is gained by time-of-flight gating with a boxcar integrator. However, because of strong fragmentation, a mass-integrated 2D spectrum is shown in Fig. 2c. Note, that except for pathway ESA2 (Supplementary Fig. 3), the photoionization step in all signal contributions scales quadratically with laser intensity. Therefore, ESA amplitudes can appear amplified in the experiment, in particular in the four-pulse scheme.

Data is taken with a laser repetition rate of 200 kHz and by integrating each delay point over 500 ms. The coherence times are scanned 0-500 fs or 0-770 fs, leading to an acquisition time of 20-30 min for one 2D map and a spectral resolution of ≤ 33 cm^{-1} (full-width half maximum). Each measurement was repeated several times and under different detection schemes to check for consistency.

The laser system comprises of an amplified oscillator (Spirit 1040-16-HE, Spectra-Physics) combined with two noncollinear optical parametric amplifiers (NOPAs, Spectra-Physics). Laser pulses 1-4 are pre-compensated with a prism compressor, yielding a pulse duration of 30 fs at the focusing lens in front of the detection chamber. Laser wavelengths (λ_L)

and pulse energies (E) measured at the same position were as follows: Fig. 2a, b - λ_L= 732 nm, E= 26 nJ, Fig. 3a - λ_L= 713 nm, E= 51 nJ and Fig. 2c - λ_L= 722 nm, E= 45 nJ (pulse 1-4), λ_L= 670 nm, E= 455 nJ (pulse 5). Examples for the laser spectral profile are given in Supplementary Fig. 4.

Supplementary Note 2: Target density:

The density of molecule-containing droplets in the interaction volume is estimated being $\leq 10^7 cm^{-3}$ [4], which corresponds to OD $\approx 10^{-11}$. The OD was estimated for the Rb_2 $a^3\Sigma_u^+ \rightarrow (1)^3\Pi_g$ transition and available dipole moments, taken from Ref. 5. Note, that in HENDI, the target density is determined by the pick-up statistic of the doping process and similar densities would be obtained for other complexes (for example molecular aggregates[6]).

Supplementary Note 3: Rb_2 analysis:

The Rb_2 triplet PECs, transition dipole moments (TDMs), and corresponding Franck-Condon factors (FCFs) are taken from our calculations reported in Ref. 27. For the sake of simplicity, the SO coupling in the $(1)^3\Pi_g$ state, leading to the SO manifold of states labelled with standard Hund's case c notation $\Omega = 0_g^\pm, 1_g, 2_g$, was included by applying a constant fine structure shift (with respect to the nuclear distance) to yield the spectra of the transition between the $a^3\Sigma_u^+$ and the $0_g^\pm$ and 2_g vibrational levels. This approximation is well satisfied as the SO corrections show little variation over the relevant region of nuclear distances (Fig. 2e) and has previously led to excellent agreement between theory and experiment[7,8].

For the absorption spectrum, FCFs for transitions from the $a^3\Sigma_u^+, \nu = 0$ level to all vibrational levels of the $(1)^3\Pi_g$ state were calculated (Supplementary Fig. 5) and weighted with the spectral intensity of the laser. The re-emission spectrum was determined by calculating the FCFs for all transitions from populated vibrational levels $(1)^3\Pi_g, \nu'$ to all bound levels of the

$a^3\Sigma_u^+$ state, weighted by the population probabilities of the excited states and the laser spectrum. All transitions have been broadened to account for the experimental resolution (33 cm^{-1}) and a droplet-induced broadening (94 cm^{-1}). In addition, a blue shift (115 cm^{-1}) was included in the simulation, determined from a fit of the absorption profiles. Such shifts and broadenings are common for alkali-helium droplet interactions[9].

The coherent oscillations of the ESA_1 and ESA_2 features were deduced by integrating the peak areas for each value of *T*. To exclude aliasing effects, *T* was at first scanned in fine steps (50 fs) for a short time range (2.5 ps) to determine the maximum oscillation frequency component (not shown). The resulting oscillation amplitudes have been normalized to unity. For better visibility, only the features correlated to the $(1)^3\Pi_g, \Omega = 0_g^\pm$ SO component are represented in Fig. 2d. The other components show qualitatively the same behavior.

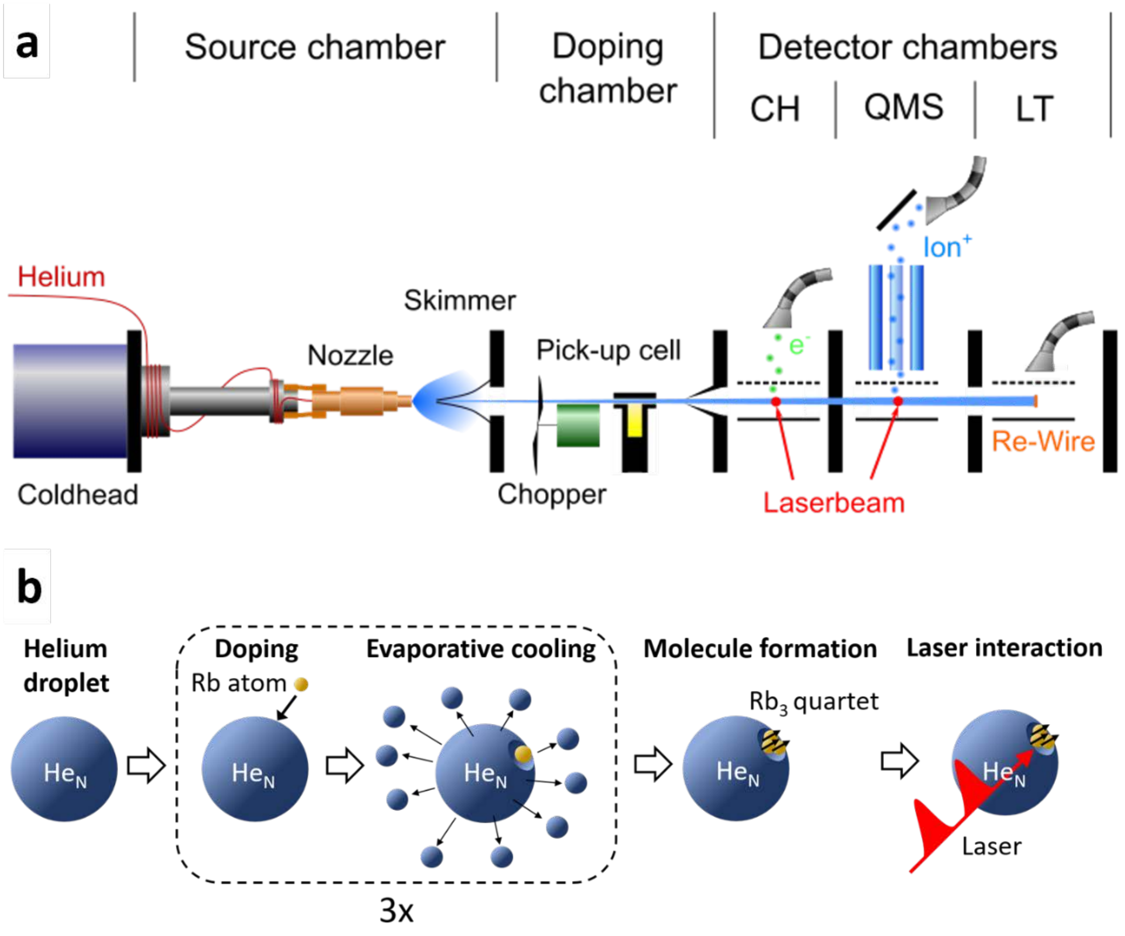

Supplementary Fig. 1 Sample preparation. **a** Vacuum apparatus used in the experiment. A supersonic beam of superfluid helium nanodroplets is generated upon adiabatic expansion, and doped with Rb atoms upon passing through a pick-up cell. Electrons/ions produced by laser ionization are detected with a channeltron detector (CH chamber). A quadrupole-mass spectrometer (QMS chamber) and a Langmuir-Taylor (LT) surface ionization detector are used for beam diagnostic/monitoring purposes. **b** Sequence of HENDI-assisted molecule formation and probing of the prepared molecule-droplet nanosystems shown for the example of Rb_3 formation in the weakly-bound quartet electronic ground state.

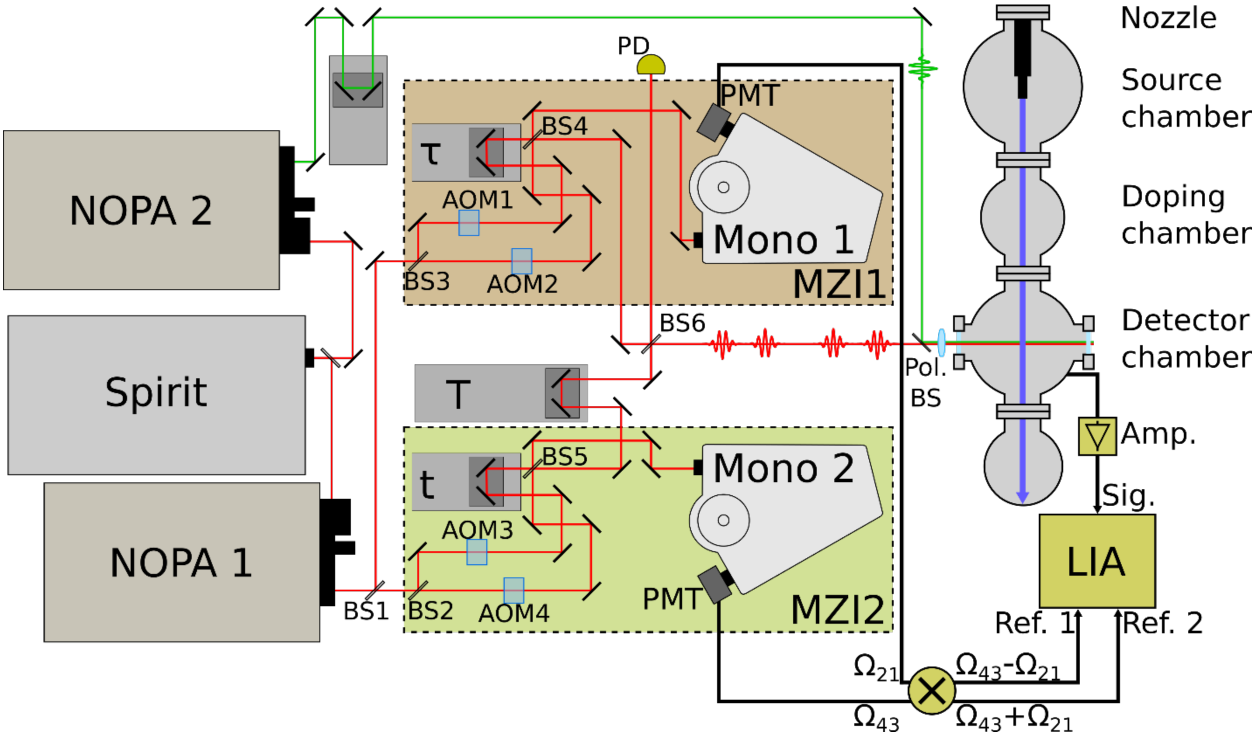

Supplementary Fig. 2 PM-2DES optical setup and signal detection. NOPA- noncollinear optical parametric amplifier, BS- beam splitter, Pol. BS- polarizing beam splitter, AOM- acousto-optical modulator, MZI- Mach-Zehnder interferometer, PD- photo diode, PMT- photo multiplier, LIA- lock-in amplifier, Amp.- amplifier, Mono- monochromator, MZI- Mach-Zehnder interferometer.

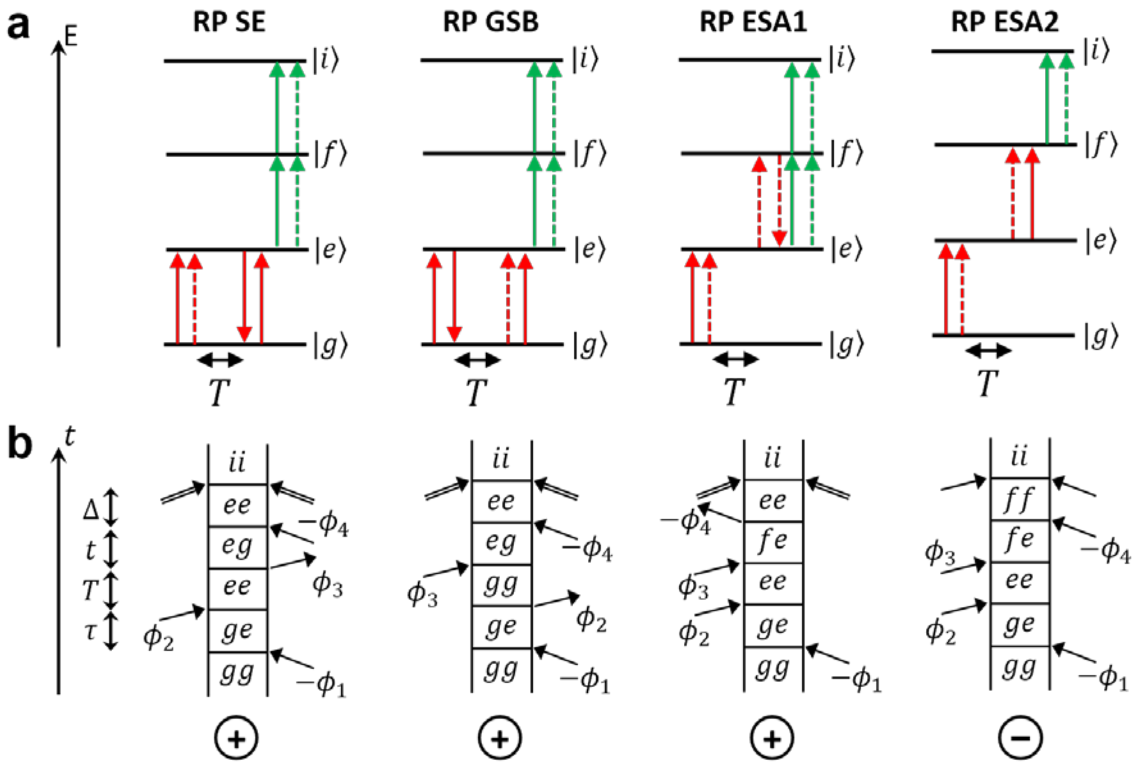

Supplementary Fig. 3 Signal pathways in Photoionization-PM-2DES experiments. **a** Simplified energy level scheme, comprised of electronic ground $|g\rangle$, single excited $|e\rangle$, double excited $|f\rangle$ and ionic state $|i\rangle$ along with the energy pathways as excited in the experiment and isolated in the lock-in detection (shown here for the example of RP signal contributions). Red-phase-modulated pulses, green- photoionization. Solid/dashed- interactions on the ket-/bra-side of the density matrix. **b** Corresponding double-sided Feynman diagrams. Common notation is used in the Feynman diagrams: Time evolves from bottom to top. Each entry denotes an element of the density matrix $|n\rangle\langle m|$. Arrows indicate the light-matter interaction leading to de-/excitation of the system. Double-arrows indicate two simultaneous interactions. ϕ_i indicates the phase factor imprinted onto the signal by each interaction. Plus/minus signs below each diagram indicate the phase/amplitude with which the processes contribute to the 2D spectra shown in the main text. Note, that we have shifted the phase by π to make comparison with other 2DES experiments more intuitive. In measurements using the four-pulse-scheme, it is Δ = 0 fs.

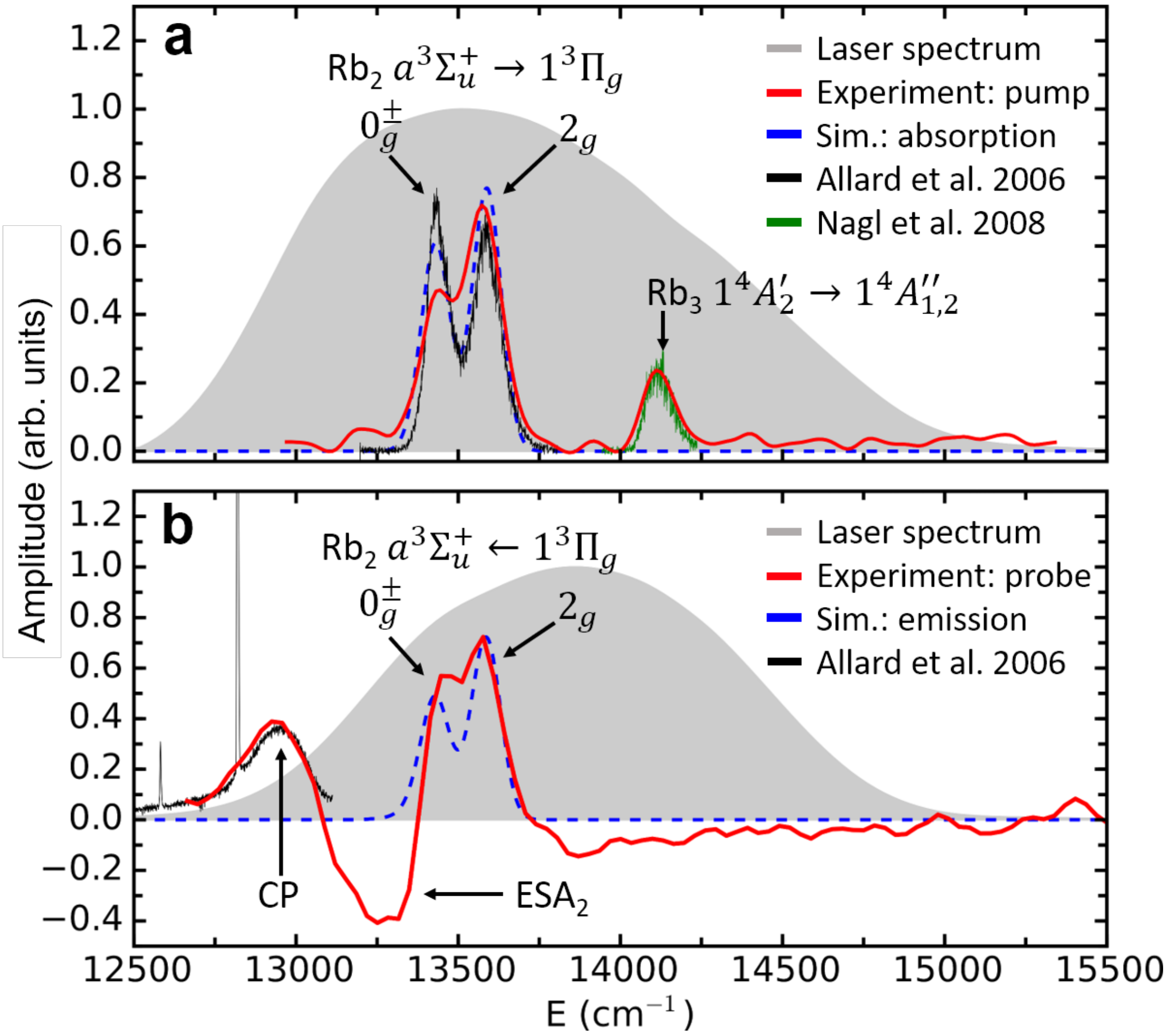

Supplementary Fig. 4 Comparison of 2DES results with high resolution steady-state laser spectroscopy. **a** Horizontal cut through Fig. 2b (obtained by integrating ω_t from 14600 to 15000 cm^{-1}) compared to cw laser spectroscopy experiments of Rb_2[7] and Rb_3[10] and a simulation of the Rb_2 absorption spectrum based on *ab initio* calculations[5,8]. **b** Vertical cut through Fig. 2c (obtained by integrating ω_τ from 13370 to 13690 cm^{-1}) compared to dispersed fluorescence of gas-phase Rb_2[7] and simulation of the Rb_2 emission spectrum based on *ab initio* calculations[5,8]. The spectral profile of the applied femtosecond laser source is shown in the background.

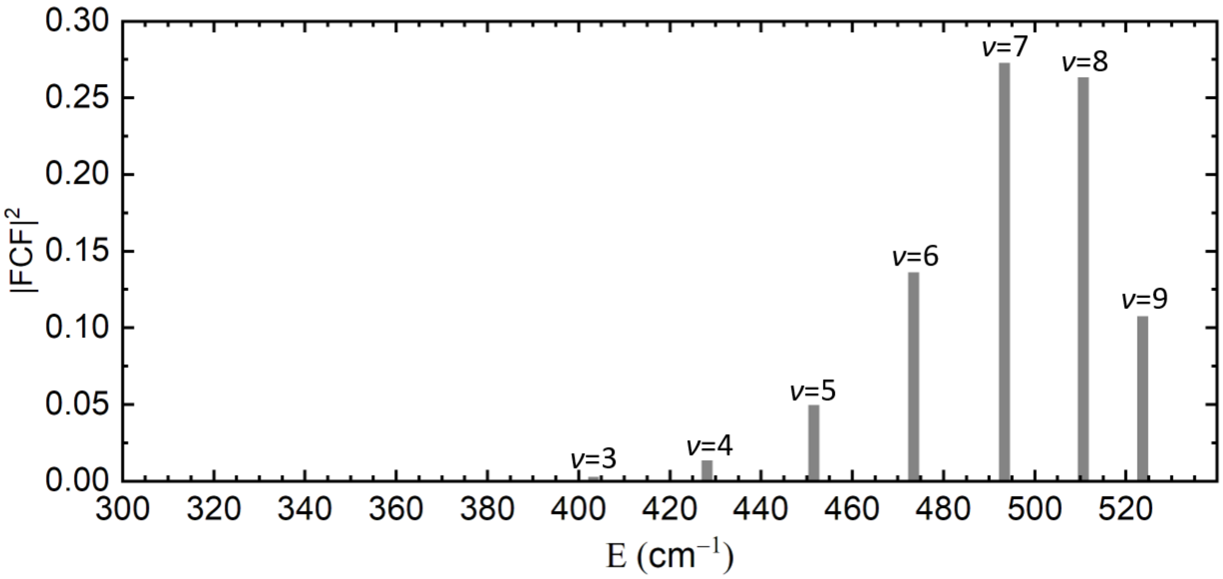

Supplementary Fig. 5 FCFs of the Rb_2 $a^3\Sigma_u^+ \rightarrow (1)^3\Pi_g$ transition. Labels indicate the vibrational levels of the $(1)^3\Pi_g$ state. The *x*-axis shows the energy of the vibrational levels relative to the 5p+5s asymptote.

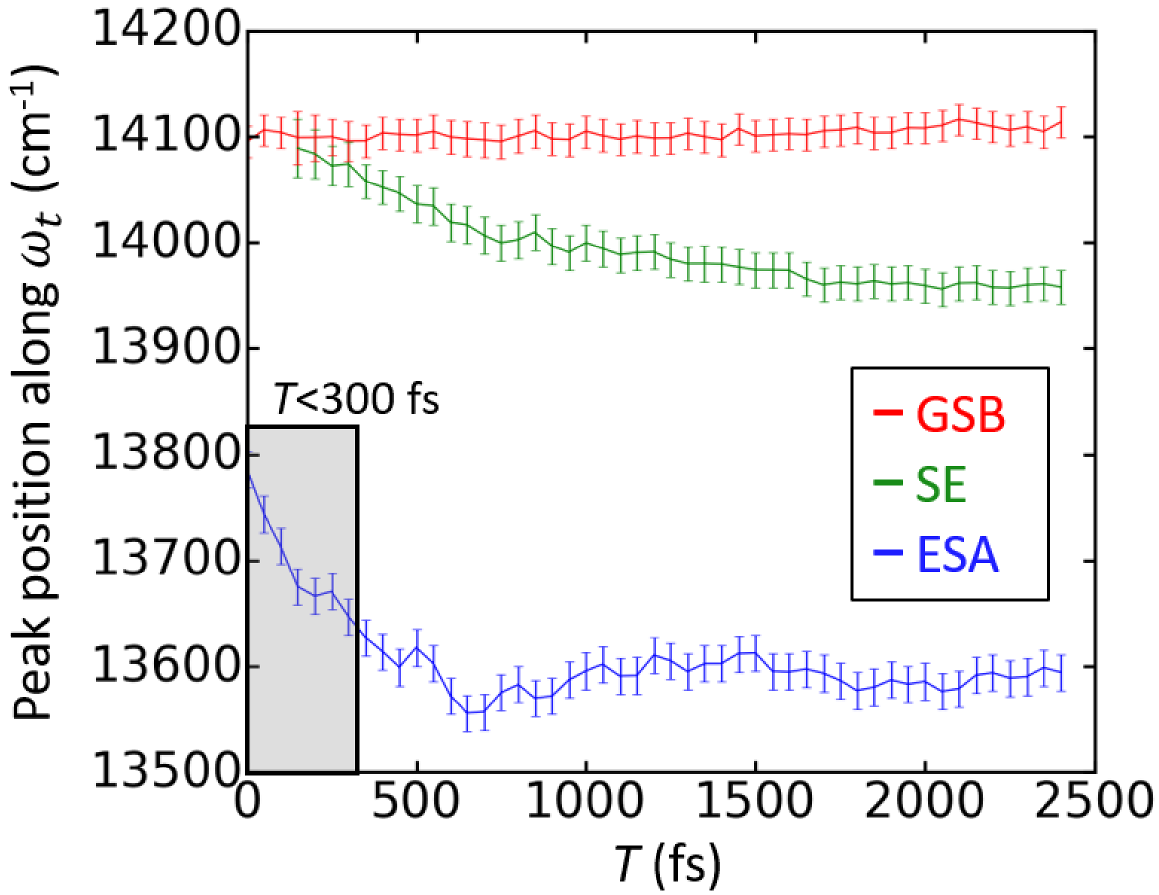

Supplementary Fig. 6 Time evolution of Rb_3 emission. Central peak positions of the Rb_3 $1^4A_2' \rightarrow 1^4A_{1,2}''$ diagonal (red) the emerging cross peak (green) and the correlated excited state absorption (ESA) peak (blue) obtained from Gaussian fits of the peak shapes along the ω_t-axis. Error bars correspond to the fitting uncertainties. Within the model of a dynamic energy shift induced by the helium environment, the first two contributions are assigned to stimulated emission (SE) and ground state bleach (GSB) pathways (label) which split in emission energy with increasing evolution time T. Note, that for $T < 300$ fs (shaded area) the position of the ESA contribution is blue shifted by an overlapping positive transient cross peak.

Supplementary References

1. Perdomo-Ortiz, A., Widom, J. R., Lott, G. A., Aspuru-Guzik, A., Marcus, A. H. Conformation and Electronic Population Transfer in Membrane-Supported Self-Assembled Porphyrin Dimers by 2D Fluorescence Spectroscopy. *J. Phys. Chem. B.* **116**, 10757–10770 (2012).

2. Fuller, F. D., Ogilvie, J. P., Experimental Implementations of Two-Dimensional Fourier Transform Electronic Spectroscopy. *Annu. Rev. Phys. Chem.* **66**, 667–690 (2015).

3. Tekavec, P. F. , Lott, G. A., Marcus, A. H., Fluorescence-detected two-dimensional electronic coherence spectroscopy by acousto-optic phase modulation. *J. Chem. Phys.* **127**, 214307 (2007).

4. Stienkemeier, F., Lehmann, K. K. Spectroscopy and dynamics in helium nanodroplets. *J. Phys. B: At. Mol. Opt. Phys.* **39**, R127 (2006).

5. Deiß, M. et al. Polarizability of ultracold Rb_2 molecules in the rovibrational ground state of $a^3\Sigma_u^+$ *New J. Phys.* **17**, 065019 (2015).

6. Roden, J., Eisfeld, A., Dvořák, M., Bünermann, O., Stienkemeier, F. Vibronic line shapes of PTCDA oligomers in helium nanodroplets. *J. Chem. Phys.* **134**, 054907 (2011).

7. Allard, O., Nagl, J., Auböck, G., Callegari, C., Ernst, W. E. Investigation of KRb and Rb_2 formed on cold helium nanodroplets. *J. Phys. B: At. Mol. Opt. Phys.* **39**, 1169-1182 (2006).

8. Bellos, M. A. et al. Formation of ultracold Rb_2 molecules in the $v'' = 0$ level of the $a^3\Sigma_u^+$ state via blue-detuned photoassociation to the $1^3\Pi_g$ state. *Phys. Chem. Chem. Phys.* **13**, 18880–18886 (2011).

9. Bünermann, O., Droppelmann, G., Hernando, A., Mayol, R., Stienkemeier, F. Unraveling the Absorption Spectra of Alkali Metal Atoms Attached to Helium Nanodroplets. *J. Phys. Chem. A.* **111**, 12684–12694 (2007).

10. Nagl, J. et al. Heteronuclear and Homonuclear High-Spin Alkali Trimers on Helium Nanodroplets. *Phys. Rev. Lett.* **100**, 063001-063004 (2008).
